# Antibody response and safety of inactivated SARS-CoV-2 vaccines in chronic hepatitis B patients with and without cirrhosis

**DOI:** 10.3389/fimmu.2023.1167533

**Published:** 2023-05-17

**Authors:** Huanhuan Cao, Yufei Huang, Chunxiu Zhong, Xingmei Liao, Wenjuan Tan, Siru Zhao, Liangxu Guo, Rong Fan

**Affiliations:** ^1^ Guangdong Provincial Key Laboratory of Viral Hepatitis Research, Nanfang Hospital, Southern Medical University, Guangzhou, China; ^2^ Guangdong Provincial Clinical Research Center for Viral Hepatitis, Nanfang Hospital, Southern Medical University, Guangzhou, China; ^3^ Key Laboratory of Infectious Diseases Research in South China, Ministry of Education, Nanfang Hospital, Southern Medical University, Guangzhou, China; ^4^ Department of Infectious Diseases, Nanfang Hospital, Southern Medical University, Guangzhou, China; ^5^ Department of Infectious Diseases, Affiliated Dongguan People’s Hospital, Southern Medical University, Dongguan, China

**Keywords:** SARS-CoV-2 vaccine, chronic hepatitis B, cirrhosis, immunogenicity, safety, liver injury

## Abstract

**Background:**

The immune response and safety of inactivated severe acute respiratory syndrome coronavirus 2 (SARS-CoV-2) vaccines among patients with chronic hepatitis B (CHB), especially those with cirrhosis, are not clear. Therefore, this study was conducted to evaluate the efficacy and safety of inactivated SARS-CoV-2 vaccines among CHB patients with and without cirrhosis.

**Patients and methods:**

A total of 643 CHB patients who received two doses of inactivated SARS-CoV-2 vaccines (BBIBP-CorV and CoronaVac) were enrolled. Serum samples were collected and tested for SARS-CoV-2 S-receptor-binding domain (S-RBD) immunoglobulin G (IgG) at enrollment. Data on adverse events (AEs) within 7 days after the second dose were obtained using a questionnaire.

**Results:**

A total of 416 non-cirrhotic and 227 cirrhotic patients were included in the analysis. Cirrhotic patients had lower antibody titers than non-cirrhotic patients after adjusting for age, sex, and time interval (2.45 *vs*. 2.60 ng/ml, *p* = 0.034). Furthermore, the study revealed that cirrhotic patients demonstrated a slower rate of seropositivity increase, with the highest rate being recorded at week 4 and reaching 94.7%. On the other hand, among non-cirrhotic patients, the seropositivity rate peak was observed at week 2 and reached 96.0%. In addition, cirrhotic patients displayed a more rapid decline in the seropositivity rate, dropping to 54.5% after ≥16 weeks, while non-cirrhotic patients exhibited a decrease to 67.2% after the same time period. The overall incidence of AEs was low (18.4%), and all AEs were mild and self-limiting. In addition, 16.0% of participants had mild liver function abnormalities, and half of them returned to normality within the next 6 months without additional therapy. The participants who experienced liver function abnormalities showed a higher seropositivity rate and antibody titer than those who did not (91.6% *vs.* 79.5%, *p *= 0.005; 2.73 *vs.* 2.41 ng/ml, *p* < 0.001).

**Conclusion:**

Cirrhotic CHB patients had lower antibody titers to inactivated SARS-CoV-2 vaccines than non-cirrhotic patients. The vaccines were generally well tolerated in both non-cirrhotic and cirrhotic CHB patient groups. Patients with abnormal liver function may have a better antibody response than those without.

## Introduction

Severe acute respiratory syndrome coronavirus 2 (SARS-CoV-2) has caused global mayhem for the last 3 years with its ongoing spread that led to the coronavirus disease 2019 (COVID-19) pandemic. Studies (1, 2) have shown that the pandemic has resulted in significant mortality and morbidity, especially in individuals with immunodeficiency originating from liver diseases, such as cirrhosis, or malignant tumors, such as breast cancer. Thus, the management of patients with chronic liver disease (CLD) during COVID-19 infection should be given more attention.

The SARS-CoV-2 vaccines were developed in a short time with scientists’ exceptional efforts, including the inactivated vaccines BBIBP-CorV (China National Biotec Group, Beijing, China) and CoronaVac (Sinovac Life Sciences, Beijing, China), the major vaccines that are currently widely administered in China. Vaccination is the primary recommended measure to prevent associated hospitalization, severe symptoms, and death (3). Given the disease heterogeneity and the multiple morbidities of patients with CLD or malignancies, vaccination-related responses, such as the seroconversion and adverse event (AE) rates, in these populations have been popular topics (4). One study in Iran showed that the inactivated SARS-CoV-2 vaccine BBIBP-CorV had a satisfactory seroconversion of 85.7% in patients with breast cancer (5). Several studies have evaluated the safety and efficacy of the vaccine in patients with CLD, but the results have been inconsistent. It was revealed that inactivated SARS-CoV-2 vaccines have achieved a favorable safety profile and efficient immunogenicity among chronic hepatitis B (CHB) patients with similar antibody seroconversion compared to healthy controls (6, 7). However, another study (8) showed poor antibody responses in 24% of CLD patients with and without cirrhosis after SARS-CoV-2 vaccination, which differed from previous findings. Ai et al. (9) also demonstrated that patients with CLD had a lower immunological response to SARS-CoV-2 vaccines than the healthy population, while the positivity rates of the SARS-CoV-2 antibodies were similar between CLD patients with and those without cirrhosis.

Taking into account the above-mentioned discrepant results, we conducted this prospective study aiming to evaluate the efficacy and safety of inactivated SARS-CoV-2 vaccines among a large sample of CHB patients with and without cirrhosis at different time points after vaccination.

## Methods

### Participants

Between June and October 2021, a total of 643 CHB outpatients with and without cirrhosis were prospectively enrolled from Nanfang Hospital, Southern Medical University, China. The inclusion criteria were as follows: a) over 18 years of age; b) were positive for hepatitis B surface antigen (HBsAg) for at least 6 months; and c) received two doses of an inactivated SARS-CoV-2 vaccine when enrolled. The exclusion criteria were as follows: a) an active or known history of SARS-CoV-2 infection; b) had close contact with confirmed SARS-CoV-2 cases; c) co-infection with HIV/hepatitis C (HCV); d) malignant tumor and other major diseases; e) pregnancy; and f) history of immunosuppressant use 6 months before the vaccination. The diagnosis of cirrhosis was made based on standard histology with or without compatible radiological findings (for detailed information, please see the **Supplementary Material**).

### Data and sample collection

At enrollment, the clinical characteristics [age, sex, and body mass index (BMI)], the presence of cirrhosis and comorbidities (diabetes and hypertension, among others), and the date of vaccination were collected. Hepatitis B virus (HBV) DNA levels, HBV serological markers, and the biochemical parameters were assessed at local laboratories. The serum samples of all participants were taken at enrollment and tested for SARS-CoV-2 S-receptor-binding domain (S-RBD) immunoglobulin G (IgG). Data on AEs within 7 days after the second dose were obtained using a questionnaire. All AEs were graded according to the scale issued by the Common Terminology Criteria for Adverse Events (CTCAE) of the U.S. National Cancer Institute (version 5.0).

### Inactivated SARS-CoV-2 vaccination

The vaccines used in the study were mainly BBIBP-CorV and CoronaVac, the major vaccines that are currently widely administered in China. The effectiveness of SARS-CoV-2 inactivated vaccines among the healthy population has been confirmed. Data from a phase III clinical study suggested that the effectiveness of two inactivated vaccines (BBIBP-CorV and CoronaVac) in preventing symptomatic COVID-19 was 72.8%–83.5% (10, 11).

### Evaluation of SARS-CoV-2 S-RBD IgG

Indirect ELISA was used to detect the IgG antibodies against the RBD of the SARS-CoV-2 spike protein (anti-S-RBD IgG) according to the manufacturer’s protocol (KE30003; Proteintech, Wuhan, China). Standard and diluted samples were added into microwells (100 µl per well), mixed well, and incubated at room temperature for 30 min. After four rounds of washes, the horseradish peroxidase (HRP)-conjugated anti-human IgG antibody was added, mixed well, and incubated for 30 min at room temperature. After four more rounds of washes, a substrate solution (3,3’,5,5′-tetramethylbenzidine, TMB) was added before adding the stop solution. Subsequently, the absorbance [optical density (OD) value] was read at 450 and 630 nm within 5 min. The measurement range was 1.56–200 ng/ml. When the OD value (450 nm) of a sample was lower than the standard value of 1, then the result was considered negative.

### Statistical analysis

For the clinical characteristics, continuous variables were expressed as medians with interquartile range (IQR) and compared using the Mann−Whitney *U* test, while categorical variables were expressed as numbers and percentages and were compared using a chi-squared test or Fisher’s exact test, as appropriate. Propensity score matching (PSM) was performed to balance the effects of potential confounding factors between subgroups. All *p*-values were two-tailed, and the level of significance was set at *p* < 0.05. Statistical Package for the Social Sciences (SPSS) version 26.0 (IBM Corp., Armonk, NY, USA) and R version 4.1.2 (R Foundation for Statistical Computing, Vienna, Austria) were used in the analysis.

## Results

### Characteristics of the enrolled patients

A total of 643 outpatients with CHB were enrolled in the analysis, including 416 (64.7%) non-cirrhotic and 227 (35.3%) cirrhotic patients. As shown in **Table 1**, the median age was 44.2 years (IQR = 38.7–51.5 years), and 83.8% of the patients were men. At enrollment, 96.6% (621/643) of the participants were receiving nucleos(t)ide analog antiviral treatment with 96.3% (618/642) HBV DNA undetectability and 20.7% (133/643) hepatitis B e-antigen (HBeAg) positivity. Fatty liver disease, hypertension, and diabetes were the most common comorbidities in the overall population. Cirrhotic patients were significantly older than non-cirrhotic patients (48.0 *vs.* 42.3 years, *p* < 0.001), with a higher percentage of men (89.0% *vs.* 81.0%, *p* = 0.009), and most of them (219/227, 96.5%) were Child−Pugh class A. Among cirrhotic patients, 7.0% (16/227) were positive for HBeAg, which was significantly lower than that in non-cirrhotic patients (117/416, 28.1%; *p* < 0.001).

**Table 1:** Clinical characteristics of the enrolled patients.

	Overall (*n* = 643)	Non-cirrhotic (*n* = 416)	Cirrhosis (*n* = 227)	*p*-value
Age (years)	44.2 (38.7–51.5)	42.3 (37.5–49.3)	48.0 (41.8–54.3)	<0.001
Sex (men), *n* (%)	539 (83.8)	337 (81.0)	202 (89.0)	0.009
BMI (kg/m2)	23.0 (21.0–24.9)	22.8 (20.8–24.7)	23.3 (21.1–25.4)	0.094
Overweight (BMI ≥24 kg/m2), *n*, %	248 (38.9)	154 (37.4)	94 (41.6)	0.296
HBeAg positivity, *n* (%)	133 (20.7)	117 (28.1)	16 (7.0)	<0.001
HBV DNA undetectability, *n* (%)	618 (96.3)	393 (94.7)	225 (99.1)	0.005
Interval between second vaccination and blood collection (weeks)	7.4 (4.0–12.0)	7.9 (4.3–12.4)	6.7 (3.7–10.4)	0.002
<4 weeks, *n* (%)	155 (24.1)	88 (21.2)	67 (29.5)	0.018
Type of inactivated vaccine				0.720
BBIBP-CorV, *n* (%)	365 (56.8)	240 (57.7)	125 (55.1)	–
CoronaVac, *n* (%)	274 (42.6)	173 (41.6)	101 (44.5)	–
Others, *n* (%)	4 (0.6)	3 (0.7)	1 (0.4)	–
ALB (g/L)	46.5 (44.6–48.3)	46.8 (44.8–48.5)	46.1 (44.2–47.9)	0.007
Child–Pugh classification				
A, *n* (%)			219 (96.5)	–
B, *n* (%)			7 (3.1)	–
C, *n* (%)			1 (0.3)	–
Diabetes mellitus, *n* (%)	31 (4.8)	11 (2.6)	20 (8.8)	<0.001
Hypertension, *n* (%)	35 (5.4)	20 (4.8)	15 (6.6)	0.336
Fatty liver disease, *n* (%)	274 (42.6)	198 (47.6)	76 (33.5)	0.001

Data displayed are the median (interquartile range) and number (percentage).

BMI, body mass index; ALB, albumin; HBeAg, hepatitis B e-antigen.

A total of 365 (56.8%) and 274 (42.6%) patients received the BBIBP-CorV and CoronaVac vaccines, respectively. The median interval between the second vaccination and blood collection in the overall population was 7.4 weeks (IQR = 4.0–12.0), while the corresponding intervals in the non-cirrhotic and cirrhotic patient groups were 7.9 and 6.7 weeks, respectively (*p* = 0.002). A total of 24.1% (155/643) of the serum samples were collected within 4 weeks after vaccination. According to the time interval between vaccination and blood collection, we divided the patients into six time point subgroups: week 1, week 2, week 4, week 8, week 12, and week ≥16. In these subgroups, there were 39 (6.1%), 43 (6.7%), 152 (23.6%), 189 (29.4%), 140 (21.8%), and 80 (12.4%) patients (*p* = 0.03), respectively.

### Antibody response to SARS-CoV-2 vaccination

The anti-RBD IgG antibody levels were determined for all the collected blood samples. In the overall population, the positive rate of the anti-RBD IgG antibody was 82.1% (528/643), which peaked at week 4 (141/152, 92.8%) and then gradually decreased to 63.7% (51/80) at week ≥16. In addition, the mean antibody titer was 2.47 ng/ml, which peaked to 2.83 ng/ml at week 2 and decreased to 2.0 ng/ml at week ≥16.

**Figure 1:**
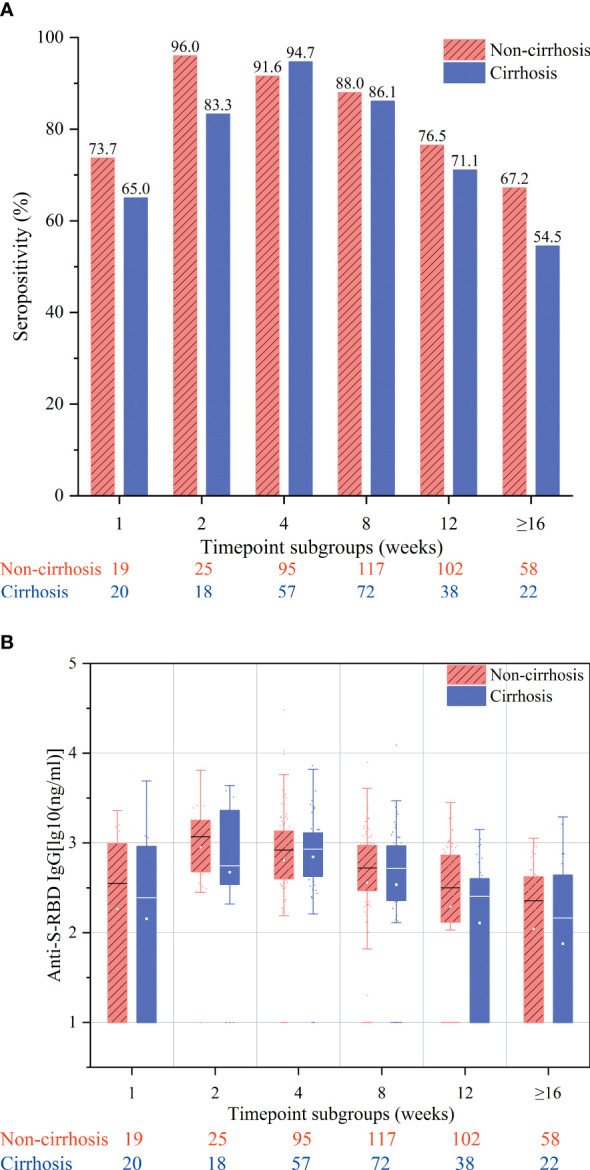
Seropositivity rates **(A)** and anti-S-receptor-binding domain (anti-S-RBD) immunoglobulin (IgG) titers **(B)** after immunization of chronic hepatitis B (CHB) patients with the inactivated SARS-CoV-2 vaccine in the week 1, 2, 4, 8, 12, and ≥16 subgroups. These subgroups included samples collected at 7 ± 2, 14 ± 2, 28 ± 7, 56 ± 14, 71–98, and ≥112 days after the second vaccination, respectively.

**Figure 2:**
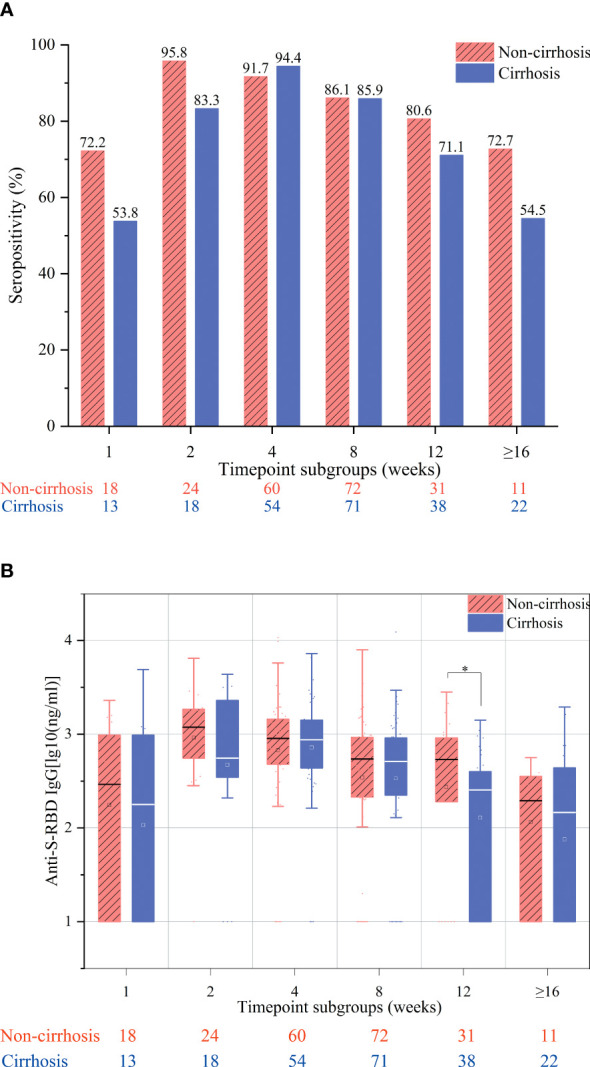
Seropositivity rates **(A)** and anti-S-receptor-binding domain (anti-S-RBD) immunoglobulin G (IgG) titers **(B)** after immunization of chronic hepatitis B (CHB) patients with the inactivated SARS-CoV-2 vaccine in the week 1, 2, 4, 8, 12, and ≥16 subgroups after adjusting for age, sex, and interval between vaccination and blood collection. These subgroups included samples collected at 7 ± 2, 14 ± 2, 28 ± 7, 56 ± 14, 71–98, and ≥112 days after the second vaccination, respectively. *p < 0.05.

The overall antibody response to vaccination was compared between cirrhotic and non-cirrhotic patients, with the results showing that the antibody rates and titers were not significantly different between these two patient groups (82.9% *vs.* 80.6%, *p* = 0.464; 2.49 *vs.* 2.45 ng/ml, *p* = 0.727). However, cirrhotic patients demonstrated a slower rate of seropositivity increase, with the highest rate being recorded at week 4 (94.7%); for non-cirrhotic patients, the seropositivity rate peak was observed at week 2 (96.0%) (**Figure 1A**). In addition, cirrhotic patients appeared to have a more rapid decline in the seropositivity rate at the ≥16-week time point, with a decrease to 54.5% compared to 67.2% in non-cirrhotic patients (*p* = 0.292). In general, the levels of anti-RBD IgG antibodies were numerically lower in cirrhotic than in non-cirrhotic patients at each time point (**Figure 1B**). After adjusting for age, sex, and time point using PSM, the overall antibody titers were significantly lower in cirrhotic than in non-cirrhotic patients (2.45 *vs.* 2.60 ng/ml, *p* = 0.034), and the trend in the change among each time point subgroup was similar to that of the overall population (**Figures 2A, B** **)**.

**Figure 3:**
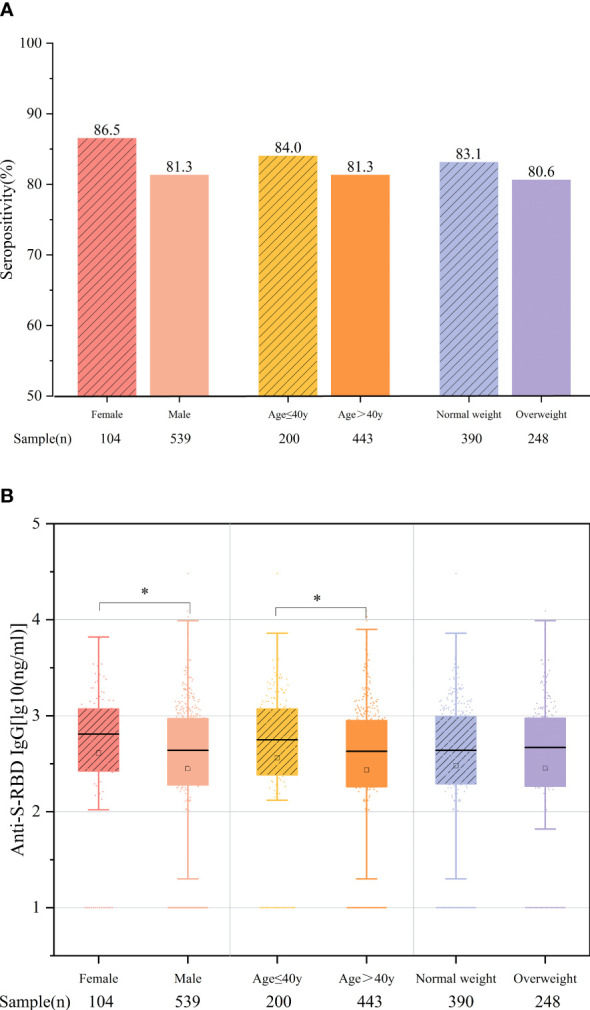
Comparison of the antibody responses according to age, sex, and body mass index (BMI). **(A)** Seropositivity rates. **(B)** Anti-S-receptor-binding domain (anti-S-RBD) immunoglobulin G (IgG) titers. *p < 0.05.

Moreover, we further compared the antibody response to vaccination according to age, sex, and BMI. The time intervals between vaccination and blood collection of the age and BMI subgroups were comparable, while those of gender were different (*p* = 0.03), which may have resulted from the difference in the morbidity rates between men and women in the CHB population. The results showed that the overall antibody titers were significantly higher in female patients aged ≤40 years than in male patients aged >40 years (women *vs.* men: 2.61 *vs.* 2.45 ng/ml, *p* = 0.024; ≤40 *vs.* >40 years: 2.56 *vs.* 2.44 ng/ml, *p* = 0.011). Between patients with normal weight (BMI <24 kg/m^2^) and those who were overweight (BMI ≥24 kg/m^2^), the antibody rates and titers were comparable (83.1% *vs.* 80.6%, *p* = 0.434; 2.48 *vs.* 2.46 ng/ml, *p* = 0.859) (**Figures 3A, B** **)**. The antibody response at different time points of the above subgroups is shown in **Supplementary Figures S1**-**S3**.

### Vaccine safety

Among the overall vaccinated population, both non-cirrhotic and cirrhotic patients tolerated the vaccines well. As shown in **Table 2**, the overall incidence of AEs within 7 days after the second dose was 18.4% (118/643) and was similar between non-cirrhotic and cirrhotic patients (19.0% *vs.* 17.2%, *p* = 0.571). All AEs were mild (grade 1) and self-limiting, and no grade 3 adverse reactions were recorded. The most common local and systemic AEs were pain (70/643, 10.9%) and fatigue (36/643, 5.6%). Moreover, we also observed that 103 of the 643 patients (16.0%) had liver function abnormalities, which were defined as any of the following parameters increasing over the upper limit of normal: alanine aminotransferase (ALT), aspartate aminotransferase (AST), total bilirubin (TB), and direct bilirubin (DB). Of the patients with abnormal liver function, 95.1% (98/103) were HBV DNA negative, while 11.6% (12/103) had fatty liver disease, which was similar to the overall population. A total of 56.3% (58/103) of these patients had normal liver function within the previous 6 months. Among the 77 patients who were re-evaluated for liver function within the next 6 months, 42.9% (33/77) had normalized liver function without receiving additional treatment, while 57.1% (44/77) had mild liver function abnormalities. Detailed information about the patients with abnormal liver function is shown in **Supplementary Table S1**.

**Table 2:** Adverse reactions within 7 days after severe acute respiratory syndrome coronavirus 2 (SARS-CoV-2) vaccination.

	Overall (*n* = 643)	Non-cirrhotic (*n* = 416)	Cirrhosis (*n* = 227)	*p*-value
Any	118 (18.4%)	79 (19.0%)	39 (17.2%)	0.571
Injection site adverse reactions				
Pain	70 (10.9%)	46 (11.1%)	24 (10.6%)	0.850
Swelling	6 (0.9%)	5 (1.2%)	1 (0.4%)	0.596
Redness	1 (0.2%)	1 (0.2%)	0 (0.0%)	1.000
Itching	1 (0.2%)	1 (0.2%)	0 (0.0%)	1.000
Systematic adverse reactions				
Headache	0 (0.0%)	0 (0.0%)	0 (0.0%)	–
Muscle pain	11 (1.7%)	7 (1.7%)	4 (1.8%)	1.000
Diarrhea	1 (0.2%)	1 (0.2%)	0 (0.0%)	1.000
Cough	0 (0.0%)	0 (0.0%)	0 (0.0%)	–
Fever	0 (0.0%)	0 (0.0%)	0 (0.0%)	–
Fatigue	36 (5.6%)	25 (6.0%)	11 (4.8%)	0.540
Others	12 (1.9%)	6 (1.4%)	6 (2.6%)	0.441
Abnormality in liver function	103 (16.0%)	49 (11.8%)	54 (23.8%)	<0.001
ALT > ULN	31 (4.8%)	21 (5.0%)	10 (4.4%)	0.716
AST > ULN	18 (2.8%)	7 (1.7%)	11 (4.8%)	0.020
TBIL > ULN	46 (7.2%)	21 (5.0%)	25 (11.0%)	0.005
DBIL > ULN	71 (11.0%)	25 (6.0%)	46 (20.3%)	<0.001

Data displayed are the number (percentage), which represent the total number of participants who had adverse reactions.

ULN, upper limit of normal; ALT, alanine aminotransferase; AST, aspartate aminotransferase; DBIL, direct bilirubin; TBIL, total bilirubin.

**Figure 4:**
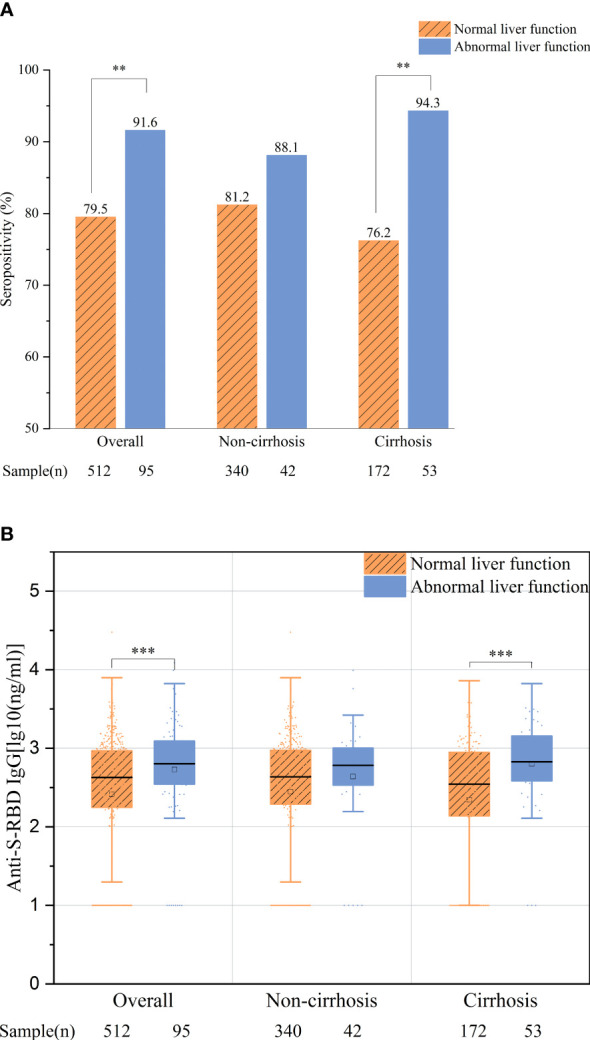
Comparison of the overall antibody response between patients with normal and abnormal liver function. **(A)** Seropositivity rates. **(B)** Anti-S-receptor-binding domain (anti-S-RBD) immunoglobulin G (IgG) titers. **p < 0.01; ***p ≤ 0.001.

More interestingly, we found that, among all the patients with negative HBV DNA and undergoing antiviral therapy (*N* = 95), those with abnormal liver function had significantly or numerically better antibody responses than those with normal liver function, regardless of the overall population (91.6% *vs.* 79.5%, *p* = 0.005; 2.73 *vs.* 2.41 ng/ml, *p* < 0.001) or the cirrhotic and non-cirrhotic patient groups (cirrhotic patients: 94.3% *vs.* 76.2%, *p* = 0.004; 2.80 *vs.* 2.34 ng/ml, *p* = 0.001; non-cirrhotic patients: 88.1% *vs.* 81.2%, *p* = 0.272; 2.64 *vs.* 2.45 ng/ml, *p* = 0.133, respectively) (**Figures 4A, B** **)**.

## Discussion

In this study, we prospectively evaluated the efficacy and safety of inactivated SARS-CoV-2 vaccines in a large sample of CHB patients with and without cirrhosis, with comprehensive clinical data collection. The results showed that cirrhotic patients were more likely to have lower levels of anti-SARS-CoV-2 antibodies and a shorter duration of antibody response than non-cirrhotic patients. The inactivated vaccines were well tolerated in both cirrhotic and non-cirrhotic patient groups due to the low incidence and mild grade of the side effects post-vaccination. These findings provide more evidence that could be useful for the management of patients with CHB during COVID-19.

Previous studies (7, 9) have reported inconsistent antibody responses among cirrhotic patients after being administered inactivated SARS-CoV-2 vaccines. In the current study, we found that cirrhotic patients had lower antibody titers than non-cirrhotic patients after adjusting for age, sex, and time interval between vaccination and blood collection. Furthermore, we divided the patients into several subgroups according to the time interval between vaccination and blood collection to compare the antibody responses between patients with and without cirrhosis at different time points. The results showed that, in addition to showing lower antibody titers at different time points, cirrhotic patients also exhibited a slower increase and a more rapid decrease in antibody levels after vaccination, indicating a shorter duration of antibody response compared with non-cirrhotic patients. A previous study (12) regarding the 23-valent pneumococcal vaccination schedule also showed that patients with end-stage liver disease had an impaired response and showed lower antibody levels and a faster decline. To our knowledge, this is the first study that evaluated the duration of antibody response after administration of an inactivated SARS-CoV-2 vaccine in cirrhotic CHB patients. It is warranted that more studies will be conducted using serial blood samples to describe in detail the dynamic change in antibody levels after vaccination. In view of the higher risks of death and severe sequelae posed by COVID-19 infection in cirrhotic patients (13, 14), it was recommended that patients with cirrhosis receive a third or even fourth dose of SARS-CoV-2 vaccine for protection. Furthermore, taking into account that half of primary liver cancer patients were from the CHB population and associated with high mortality and hospitalization rates due to COVID-19, although patients with malignancies were excluded in this paper, it was suggested that healthcare practitioners should have a more positive attitude toward the vaccination of patients with cancer (5, 15).

The impaired immunogenicity of the SARS-CoV-2 vaccine in cirrhotic patients may be attributed to the immune dysfunction state caused by liver fibrosis (16, 17). It might also be associated with the impairment of local immune surveillance function in the liver and the dysfunction of systemic immune cells (18). Compared to the healthy group, patients with cirrhosis showed a weaker and poorer T-cell response, with less INF-γ release after the second SARS-CoV-2 vaccination (19). More research is needed to further explore the underlying mechanism of the compromised response of patients with cirrhosis to SARS-CoV-2 vaccines.

We also evaluated the antibody response according to sex, age, and BMI. It was found that women and younger patients had better antibody response to vaccination than men and older patients. On the other hand, the antibody responses between normal weight and overweight patients were similar. These findings were consistent with those of previous studies regarding other vaccines. Fink et al. (20) reported that greater activation of TLR7 and the production of antibodies in female mice improved the efficacy of the influenza vaccine. In addition, another study (21) reported that more activation of T cells in female patients implied a better immune response during SARS-CoV-2 infection. Regarding the age-dependent antibody response, Walsh et al. confirmed that the antibody response to COVID-19 vaccines decreased with age, which may be associated with immunosenescence, as CD8^+^ T cells and CD4^+^ naive cells decrease with age (22, 23).

There have been several reports of immune-mediated liver injury (ILI) following COVID-19 vaccination worldwide (24, 25). As mentioned in the literature review, 26.1% of patients with ILI had preexisting liver disease (25). In this study, we also observed that nearly a quarter of the patients had mild liver function abnormalities, of which 92.2% were undergoing antivirus therapy and showed HBV DNA negativity. We also found that 56.3% of the patients had normal liver function at the previous follow-up, with 42.8% returning to normal liver function within the next 6 months without additional treatment. These liver injury cases could have been induced by vaccination, but the influence of other factors, such as non-alcoholic fatty liver disease (NAFLD), other drugs, or alcohol consumption, still cannot be excluded. More interestingly, we also found that patients who experienced abnormal liver function had a better antibody response than those without liver injury. A previous study also reported a similar finding, where participants with adverse reactions after the second dose of a SARS-CoV-2 vaccine had better antibody titers (26). Hence, it was hypothesized that mild liver injury is partially related to humoral immunity after administration of an inactivated vaccine (26, 27). Further studies with long-term monitoring of the serum indicator of the liver function of patients after SARS-CoV-2 vaccine administration are essential in order to draw conclusions on the potential link between liver injury and humoral immunity.

Regarding the safety of inactivated SARS-CoV-2 vaccines, this study and previous studies demonstrated that these vaccines were well tolerated by both cirrhotic and non-cirrhotic patients (6, 7). Recent studies have revealed that the vaccination rate in patients with cirrhosis was significantly lower than that in the general population worldwide, especially in patients with decompensated cirrhosis, and only 37.1% of these patients received at least one dose of SARS-CoV-2 vaccine (27, 28). The main reasons for these patients remaining unvaccinated were the lack of positive advice from medical providers and the fear of negative effects from the vaccine (27). Based on the above evidence, we recommend that SARS-CoV-2 vaccination be popularized among patients with CHB and compensated cirrhosis.

This study had a few limitations. Firstly, serial blood samples from the same individual were not collected in the current study, which made it impossible to accurately describe the dynamic changes of antibodies after vaccination. Secondly, as the primary objective of this study was to compare the antibody responses between patients with and without cirrhosis after vaccination, healthy volunteers were not enrolled in this study. However, it was found that the response rate of non-cirrhotic CHB patients was similar to the previously reported response of healthy people to vaccines (11, 29). Thirdly, only the anti-S-RBD IgG antibodies were analyzed to assess the humoral response to the vaccine due to the lack of detection of neutralizing antibodies and T-cell function tests. Fourthly, because most of the cirrhotic patients enrolled in our study were Child−Pugh class A, we were not able to further compare the antibody levels of these patients to those in different classes. Lastly, the sample size in our study may be relatively small for subgroup analysis, which limited conducting an extensive analysis of the different responses to the vaccine between cirrhotic and non-cirrhotic patients.

In conclusion, cirrhotic CHB patients had a lower antibody response to inactivated SARS-CoV-2 vaccines than non-cirrhotic patients. Vaccines were generally well tolerated by CHB patients with and without cirrhosis. Patients who experienced abnormal liver function may have a better antibody response than those who did not, which warrants further investigation.

## Data availability statement

The raw data supporting the conclusions of this article will be made available by the authors, without undue reservation.

## Ethics statement

The studies involving human participants were reviewed and approved by the Ethics Committee of Nanfang Hospital. The patients/participants provided written informed consent to participate in this study.

## Author contributions

RF: Concept and design, critical revision of the manuscript, and supervision. HC, YH, CZ, XL, WT, SZ, and LG: Data collection. HC, YH, and CZ: Data analysis. HC and YH: Drafting of the manuscript. All authors contributed to the article and approved the submitted version.
