# Supplementary material for: Antibody response and safety of inactivated SARS-CoV-2 vaccines in chronic hepatitis B patients with and without cirrhosis
Source: Front Immunol. 2023 May 17;14:1167533. doi: 10.3389/fimmu.2023.1167533 (PMC10230951; doi:10.3389/fimmu.2023.1167533)
Supplement: Supplementary file 1 [file DataSheet_1.docx]

**Supplementary material**

The diagnosis of cirrhosis was made based on one or more of the following criteria, a) Histologically cirrhosis; b) Meeting one or more of the following criteria on the basis of excluding of non-cirrhotic portal hypertension: 1)history of ascites; 2) history of hepatic encephalopathy; 3) history of varicose veins rupture and bleeding; c) Meeting two or more of the following criteria: 1) Imaging of cirrhosis or portal hypertension; 2) Platelet (PLT) < 100 × 10^9/L, without any other reasons; 3) Fibrosis score measured by FibroScan is larger than 12Kpa with the Serum ALT<50 U/L; 4) Gastroscopy shows varicose veins of the esophagus.

**Supplementary table 1.** Detailed information of laboratory data on patients with abnormal liver function

|  | T-6 months | | | |  | Liver function at enrolled (T) | | | |  | T+6 months | | | |
| --- | --- | --- | --- | --- | --- | --- | --- | --- | --- | --- | --- | --- | --- | --- |
| Patients ID | ALT | AST | TIBL | DBIL |  | ALT | AST | TIBL | DBIL |  | ALT | AST | TIBL | DBIL |
| 1 | 38.0 | 34.0 | 9.9 | 4.8 |  | 54.0 | 38.0 | 11.2 | 4.0 |  | 48.0 | 35.0 | 11.4 | 4.3 |
| 2 | 27.0 | 20.0 | 20.5 | 6.8 |  | 35.0 | 28.0 | 33.2 | 8.1 |  | 24.0 | 21.0 | 18.8 | 5.4 |
| 3 | 51.0 | 25.0 | 9.4 | 3.0 |  | 325.0 | 104.0 | 21.6 | 5.4 |  | 232.0 | 65.0 | 16.9 | 5.4 |
| 4 | 11.0 | 18.0 | 27.3 | 6.9 |  | 12.0 | 18.0 | 39.0 | 9.5 |  | 14.0 | 19.0 | 36.2 | 12.0 |
| 5 | 32.0 | 24.0 | 25.7 | 6.8 |  | 33.0 | 21.0 | 28.0 | 7.2 |  | 32.0 | 20.0 | 23.9 | 7.1 |
| 6 | 41.0 | 21.0 | 10.3 | 3.0 |  | 70.0 | 34.0 | 9.2 | 2.7 |  | 45.0 | 29.0 | 8.0 | 2.5 |
| 7 | 19.0 | 15.0 | 19.9 | 6.0 |  | 16.0 | 13.0 | 27.7 | 7.4 |  | NA | NA | NA | NA |
| 8 | 41.0 | 22.0 | 15.8 | 4.5 |  | 73.0 | 34.0 | 14.1 | 5.1 |  | NA | NA | NA | NA |
| 9 | 29.0 | 37.0 | 13.4 | 4.7 |  | 51.0 | 32.0 | 12.4 | 3.7 |  | NA | NA | NA | NA |
| 10 | 17.0 | 19.0 | 33.1 | 7.2 |  | 17.0 | 24.0 | 39.0 | 11.1 |  | 15.0 | 18.0 | 31.4 | 9.1 |
| 11 | 201.0 | 102.0 | 11.0 | 4.6 |  | 277.0 | 119.0 | 6.6 | 3.4 |  | NA | NA | NA | NA |
| 12 | 34.0 | 18.0 | 33.1 | 10.4 |  | 34.0 | 20.0 | 22.7 | 8.1 |  | NA | NA | NA | NA |
| 13 | 21.0 | 18.0 | 17.4 | 4.9 |  | 60.0 | 30.0 | 19.5 | 5.6 |  | NA | NA | NA | NA |
| 14 | 43.0 | 29.0 | 23.0 | 7.0 |  | 41.0 | 32.0 | 28.3 | 9.1 |  | 29.0 | 26.0 | 19.2 | 5.6 |
| 15 | 12.0 | 19.0 | 22.9 | 6.2 |  | 12.0 | 15.0 | 32.1 | 8.1 |  | 19.0 | 22.0 | 25.7 | 5.8 |
| 16 | 24.0 | 20.0 | 19.7 | 8.2 |  | 17.0 | 19.0 | 29.1 | 11.0 |  | NA | NA | NA | NA |
| 17 | 20.0 | 18.0 | 35.3 | 10.0 |  | 22.0 | 17.0 | 37.2 | 12.6 |  | 19.0 | 17.0 | 33.9 | 9.5 |
| 18 | 22.0 | 22.0 | 23.7 | 8.1 |  | 24.0 | 22.0 | 37.7 | 11.8 |  | 25.0 | 23.0 | 23.1 | 7.0 |
| 19 | 19.0 | 19.0 | 19.6 | 6.5 |  | 18.0 | 19.0 | 24.0 | 8.5 |  | 36.0 | 23.0 | 21.5 | 5.6 |
| 20 | 18.0 | 24.0 | 22.1 | 6.1 |  | 12.0 | 20.0 | 27.1 | 8.7 |  | NA | NA | NA | NA |
| 21 | 18.0 | 19.0 | 14.5 | 5.2 |  | 20.0 | 18.0 | 34.6 | 10.6 |  | 17.0 | 19.0 | 10.7 | 4.7 |
| 22 | 32.0 | 24.0 | 12.6 | 4.9 |  | 51.0 | 31.0 | 7.5 | 3.8 |  | 23.0 | 21.0 | 8.2 | 3.7 |
| 23 | 52.0 | 29.0 | 16.9 | 4.9 |  | 75.0 | 37.0 | 9.5 | 3.4 |  | NA | NA | NA | NA |
| 24 | 18.0 | 24.0 | 20.5 | 6.7 |  | 74.0 | 60.0 | 20.1 | 6.7 |  | 47.0 | 31.0 | 29.1 | 9.0 |
| 25 | 77.0 | 39.0 | 4.9 | 1.5 |  | 71.0 | 42.0 | 5.9 | 2.5 |  | NA | NA | NA | NA |
| 26 | 38.0 | 23.0 | 10.9 | 3.3 |  | 55.0 | 34.0 | 7.7 | 2.1 |  | 37.0 | 21.0 | 8.1 | 3.2 |
| 27 | 61.0 | 32.0 | 15.4 | 5.0 |  | 55.0 | 32.0 | 18.6 | 5.9 |  | 89.0 | 37.0 | 18.0 | 5.7 |
| 28 | 15.0 | 26.0 | 21.2 | 5.8 |  | 16.0 | 22.0 | 28.5 | 9.0 |  | 38.0 | 32.0 | 12.7 | 4.5 |
| 29 | 19.0 | 22.0 | 9.3 | 3.1 |  | 14.0 | 18.0 | 23.3 | 8.2 |  | 19.0 | 20.0 | 11.4 | 3.5 |
| 30 | 12.0 | 14.0 | 17.6 | 5.2 |  | 23.0 | 14.0 | 26.2 | 8.4 |  | 17.0 | 18.0 | 15.7 | 5.1 |
| 31 | 21.0 | 16.0 | 9.5 | 2.5 |  | 51.0 | 27.0 | 9.3 | 2.2 |  | NA | NA | NA | NA |
| 32 | 20.0 | 22.0 | 12.6 | 3.0 |  | 69.0 | 41.0 | 17.5 | 5.8 |  | NA | NA | NA | NA |
| 33 | 25.0 | 25.0 | 22.7 | 7.1 |  | 16.0 | 13.0 | 20.2 | 8.1 |  | NA | NA | NA | NA |
| 34 | 49.0 | 26.0 | 17.5 | 6.3 |  | 55.0 | 24.0 | 14.6 | 6.2 |  | 29.0 | 19.0 | 12.9 | 5.0 |
| 35 | 28.0 | 49.0 | 9.8 | 3.9 |  | 31.0 | 80.0 | 10.3 | 5.5 |  | NA | NA | NA | NA |
| 36 | 17.0 | 18.0 | 17.2 | 4.2 |  | 15.0 | 16.0 | 27.9 | 8.3 |  | 10.0 | 15.0 | 24.6 | 7.5 |
| 37 | 18.0 | 19.0 | 12.1 | 4.2 |  | 38.0 | 26.0 | 27.4 | 9.1 |  | 50.0 | 44.0 | 26.4 | 7.3 |
| 38 | 25.0 | 23.0 | 25.7 | 7.3 |  | 27.0 | 21.0 | 29.5 | 8.4 |  | NA | NA | NA | NA |
| 39 | 35.0 | 25.0 | 8.8 | 3.1 |  | 57.0 | 34.0 | 7.7 | 3.5 |  | NA | NA | NA | NA |
| 40 | 10.0 | 15.0 | 20.5 | 8.7 |  | 14.0 | 20.0 | 27.7 | 11.5 |  | 11.0 | 15.0 | 27.4 | 10.9 |
| 41 | 15.0 | 19.0 | 15.7 | 6.1 |  | 16.0 | 17.0 | 22.7 | 9.0 |  | 18.0 | 20.0 | 11.4 | 4.9 |
| 42 | 19.0 | 24.0 | 20.3 | 7.4 |  | 18.0 | 21.0 | 21.4 | 9.4 |  | 19.0 | 21.0 | 18.4 | 6.9 |
| 43 | 33.0 | 21.0 | 14.0 | 5.5 |  | 54.0 | 25.0 | 10.7 | 4.3 |  | NA | NA | NA | NA |
| 44 | 38.0 | 28.0 | 8.1 | 3.4 |  | 77.0 | 40.0 | 6.2 | 2.9 |  | 47.0 | 32.0 | 6.3 | 3.2 |
| 45 | 27.0 | 19.0 | 30.6 | 8.6 |  | 26.0 | 18.0 | 29.8 | 7.3 |  | 41.0 | 23.0 | 30.7 | 7.3 |
| 46 | 27.0 | 19.0 | 19.8 | 8.5 |  | 23.0 | 18.0 | 18.8 | 10.0 |  | 44.0 | 25.0 | 15.7 | 7.8 |
| 47 | 39.0 | 24.0 | 14.3 | 5.9 |  | 68.0 | 45.0 | 34.0 | 11.1 |  | 29.0 | 22.0 | 27.3 | 10.6 |
| 48 | 21.0 | 14.0 | 9.5 | 3.2 |  | 18.0 | 11.0 | 30.7 | 9.0 |  | 29.0 | 16.0 | 23.5 | 7.4 |
| 49 | 77.0 | 34.0 | 10.4 | 3.9 |  | 70.0 | 40.0 | 10.6 | 4.2 |  | 51.0 | 29.0 | 10.3 | 4.0 |
| 50 | 20.0 | 25.0 | 25.9 | 10.1 |  | 28.0 | 30.0 | 29.8 | 11.1 |  | 21.0 | 26.0 | 25.4 | 10.3 |
| 51 | 41.0 | 46.0 | 43.2 | 16.3 |  | 31.0 | 39.0 | 55.1 | 20.5 |  | 26.0 | 32.0 | 44.3 | 16.1 |
| 52 | 10.0 | 20.0 | 29.9 | 19.1 |  | 11.0 | 28.0 | 28.7 | 10.0 |  | 10.0 | 24.0 | 19.7 | 7.6 |
| 53 | 21.0 | 23.0 | 21.8 | 5.0 |  | 30.0 | 28.0 | 25.6 | 9.9 |  | NA | NA | NA | NA |
| 54 | 85.0 | 42.0 | 12.5 | 4.3 |  | 53.0 | 30.0 | 17.9 | 4.7 |  | NA | NA | NA | NA |
| 55 | 39.0 | 49.0 | 30.6 | 11.1 |  | 29.0 | 37.0 | 28.7 | 12.0 |  | 39.0 | 38.0 | 29.7 | 12.2 |
| 56 | 28.0 | 31.0 | 28.9 | 9.6 |  | 43.0 | 40.0 | 34.0 | 13.9 |  | 40.0 | 37.0 | 29.4 | 8.3 |
| 57 | 26.0 | 23.0 | 31.9 | 10.3 |  | 28.0 | 25.0 | 25.7 | 9.9 |  | 18.0 | 19.0 | 28.7 | 9.9 |
| 58 | 34.0 | 57.0 | 39.4 | 16.9 |  | 30.0 | 53.0 | 34.9 | 15.1 |  | NA | NA | NA | NA |
| 59 | 12.0 | 19.0 | 27.9 | 8.0 |  | 8.0 | 17.0 | 33.6 | 10.5 |  | 13.0 | 20.0 | 20.2 | 6.5 |
| 60 | 18.0 | 20.0 | 14.3 | 5.1 |  | 106.0 | 39.0 | 29.8 | 15.0 |  | NA | NA | NA | NA |
| 61 | 8.0 | 16.0 | 52.9 | 15.8 |  | 6.0 | 17.0 | 54.8 | 16.7 |  | 11.0 | 21.0 | 31.8 | 13.8 |
| 62 | 34.0 | 24.0 | 14.0 | 3.1 |  | 69.0 | 29.0 | 20.1 | 6.5 |  | 52.0 | 24.0 | 27.3 | 7.2 |
| 63 | 41.0 | 40.0 | 18.5 | 6.2 |  | 45.0 | 42.0 | 12.9 | 5.3 |  | 55.0 | 35.0 | 12.2 | 4.3 |
| 64 | 51.0 | 29.0 | 17.2 | 7.0 |  | 57.0 | 37.0 | 25.4 | 8.7 |  | 24.0 | 21.0 | 24.3 | 8.8 |
| 65 | 41.0 | 69.0 | 68.4 | 37.8 |  | 36.0 | 65.0 | 62.0 | 34.4 |  | 49.0 | 90.0 | 73.9 | 41.9 |
| 66 | 20.0 | 34.0 | 21.0 | 9.3 |  | 27.0 | 43.0 | 43.4 | 18.5 |  | 24.0 | 38.0 | 27.5 | 12.0 |
| 67 | 16.0 | 21.0 | 23.6 | 6.0 |  | 27.0 | 24.0 | 25.3 | 8.9 |  | 18.0 | 21.0 | 16.4 | 5.9 |
| 68 | 34.0 | 36.0 | 21.5 | 8.2 |  | 31.0 | 36.0 | 24.1 | 10.9 |  | 26.0 | 29.0 | 21.7 | 8.1 |
| 69 | 25.0 | 25.0 | 23.6 | 6.8 |  | 38.0 | 37.0 | 20.2 | 8.6 |  | 27.0 | 25.0 | 21.7 | 6.9 |
| 70 | 41.0 | 51.0 | 30.8 | 12.5 |  | 42.0 | 59.0 | 43.9 | 16.1 |  | 31.0 | 42.0 | 40.9 | 17.4 |
| 71 | 21.0 | 24.0 | 20.8 | 8.9 |  | 16.0 | 21.0 | 23.7 | 12.1 |  | 31.0 | 39.0 | 54.3 | 18.5 |
| 72 | 27.0 | 26.0 | 18.3 | 5.7 |  | 26.0 | 22.0 | 24.4 | 10.1 |  | 20.0 | 23.0 | 11.3 | 4.2 |
| 73 | 16.0 | 26.0 | 35.4 | 9.8 |  | 18.0 | 26.0 | 28.8 | 9.0 |  | NA | NA | NA | NA |
| 74 | 44.0 | 28.0 | 16.1 | 5.7 |  | 68.0 | 44.0 | 23.1 | 7.8 |  | 61.0 | 34.0 | 18.5 | 6.9 |
| 75 | 29.0 | 27.0 | 25.5 | 6.3 |  | 28.0 | 26.0 | 27.1 | 11.0 |  | 35.0 | 30.0 | 19.4 | 7.1 |
| 76 | 29.0 | 36.0 | 41.6 | 13.2 |  | 33.0 | 42.0 | 55.8 | 17.7 |  | 32.0 | 33.0 | 51.3 | 15.6 |
| 77 | 14.0 | 25.0 | 26.5 | 10.7 |  | 16.0 | 24.0 | 33.1 | 15.2 |  | 15.0 | 28.0 | 24.5 | 11.6 |
| 78 | 52.0 | 45.0 | 10.8 | 4.7 |  | 205.0 | 153.0 | 7.5 | 4.1 |  | 137.0 | 127.0 | 5.5 | 3.3 |
| 79 | 44.0 | 32.0 | 12.4 | 3.9 |  | 64.0 | 41.0 | 17.0 | 6.0 |  | 50.0 | 40.0 | 13.4 | 4.8 |
| 80 | 30.0 | 43.0 | 42.0 | 15.9 |  | 26.0 | 39.0 | 63.3 | 20.5 |  | 19.0 | 28.0 | 40.2 | 15.5 |
| 81 | 14.0 | 22.0 | 15.0 | 5.2 |  | 13.0 | 19.0 | 23.7 | 9.1 |  | NA | NA | NA | NA |
| 82 | 23.0 | 22.0 | 25.1 | 8.3 |  | 21.0 | 20.0 | 29.9 | 9.3 |  | NA | NA | NA | NA |
| 83 | 82.0 | 73.0 | 17.6 | 7.9 |  | 89.0 | 58.0 | 16.6 | 7.2 |  | 54.0 | 44.0 | 22.7 | 8.3 |
| 84 | 40.0 | 23.0 | 22.7 | 6.9 |  | 55.0 | 38.0 | 24.6 | 11.1 |  | 29.0 | 17.0 | 22.5 | 10.4 |
| 85 | 21.0 | 28.0 | 24.7 | 7.5 |  | 14.0 | 26.0 | 19.6 | 8.5 |  | 17.0 | 28.0 | 22.8 | 8.6 |
| 86 | 22.0 | 19.0 | 20.9 | 7.7 |  | 19.0 | 21.0 | 29.3 | 9.9 |  | 18.0 | 19.0 | 28.4 | 10.4 |
| 87 | 40.0 | 21.0 | 16.4 | 5.3 |  | 52.0 | 24.0 | 11.6 | 3.9 |  | 68.0 | 36.0 | 6.9 | 3.2 |
| 88 | 21.0 | 30.0 | 15.5 | 5.2 |  | 29.0 | 36.0 | 17.6 | 8.5 |  | 21.0 | 28.0 | 14.2 | 6.8 |
| 89 | 15.0 | 28.0 | 30.0 | 10.5 |  | 14.0 | 26.0 | 29.4 | 11.7 |  | 15.0 | 30.0 | 27.7 | 11.2 |
| 90 | 22.0 | 23.0 | 18.8 | 6.5 |  | 30.0 | 39.0 | 18.2 | 8.9 |  | 106.0 | 131.0 | 55.9 | 43.6 |
| 91 | 17.0 | 24.0 | 42.4 | 11.3 |  | 15.0 | 20.0 | 38.2 | 12.8 |  | 19.0 | 25.0 | 43.3 | 12.9 |
| 92 | 25.0 | 35.0 | 11.8 | 5.2 |  | 26.0 | 37.0 | 19.2 | 8.3 |  | 31.0 | 39.0 | 27.8 | 11.9 |
| 93 | 23.0 | 22.0 | 15.5 | 5.1 |  | 21.0 | 18.0 | 25.4 | 9.1 |  | 37.0 | 29.0 | 25.7 | 9.2 |
| 94 | 32.0 | 36.0 | 14.2 | 5.4 |  | 23.0 | 24.0 | 26.7 | 9.1 |  | 19.0 | 25.0 | 12.2 | 4.2 |
| 95 | 29.0 | 21.0 | 23.2 | 10.3 |  | 30.0 | 21.0 | 21.2 | 9.8 |  | 28.0 | 19.0 | 22.5 | 11.5 |
| 96 | 31.0 | 31.0 | 35.1 | 11.7 |  | 28.0 | 28.0 | 28.0 | 9.0 |  | NA | NA | NA | NA |
| 97 | 27.0 | 36.0 | 20.6 | 7.8 |  | 31.0 | 47.0 | 23.4 | 9.0 |  | 28.0 | 37.0 | 19.5 | 6.5 |
| 98 | 14.0 | 22.0 | 25.6 | 8.0 |  | 16.0 | 23.0 | 31.1 | 8.2 |  | 25.0 | 48.0 | 26.6 | 2.3 |
| 99 | 27.0 | 31.0 | 17.7 | 6.6 |  | 23.0 | 25.0 | 23.1 | 9.3 |  | 32.0 | 28.0 | 17.2 | 6.9 |
| 100 | 62.0 | 39.0 | 10.4 | 4.2 |  | 33.0 | 25.0 | 24.2 | 9.2 |  | 41.0 | 28.0 | 18.9 | 7.5 |
| 101 | 27.0 | 23.0 | 20.1 | 5.9 |  | 25.0 | 22.0 | 25.5 | 9.1 |  | NA | NA | NA | NA |
| 102 | 17.0 | 18.0 | 19.2 | 7.7 |  | 24.0 | 25.0 | 27.8 | 10.3 |  | 16.0 | 20.0 | 29.8 | 11.9 |
| 103 | 10.0 | 23.0 | 30.5 | 12.3 |  | 11.0 | 21.0 | 18.7 | 8.8 |  | 13.0 | 23.0 | 19.0 | 8.6 |

Note: T-6 months, liver function data within six months before the sample collection; T, liver function at the patients enrolled; T+6 months, liver function data within six months after the sample collection; ALT, alanine aminotransferase, U/L; AST, aspartate aminotransferase, U/L; DBIL, direct bilirubin, μmol/L; TBIL, total bilirubin, μmol/L; NA, not available.

**Supplementary Figure 1.** The comparison of antibody response in week 1, 2, 4, 8, 12, ≥16 subgroups according to sex. Seropositivity rates (A) and the anti-S-RBD IgG titer (B) in female and male subgroups. The week 1, 2, 4, 8, 12, ≥16 subgroups included the samples collected at 7±2 days, 14±2 days, 28±7 days, 56±14 days, 71-98 days and ≥112 days after 2nd vaccination, respectively.

| 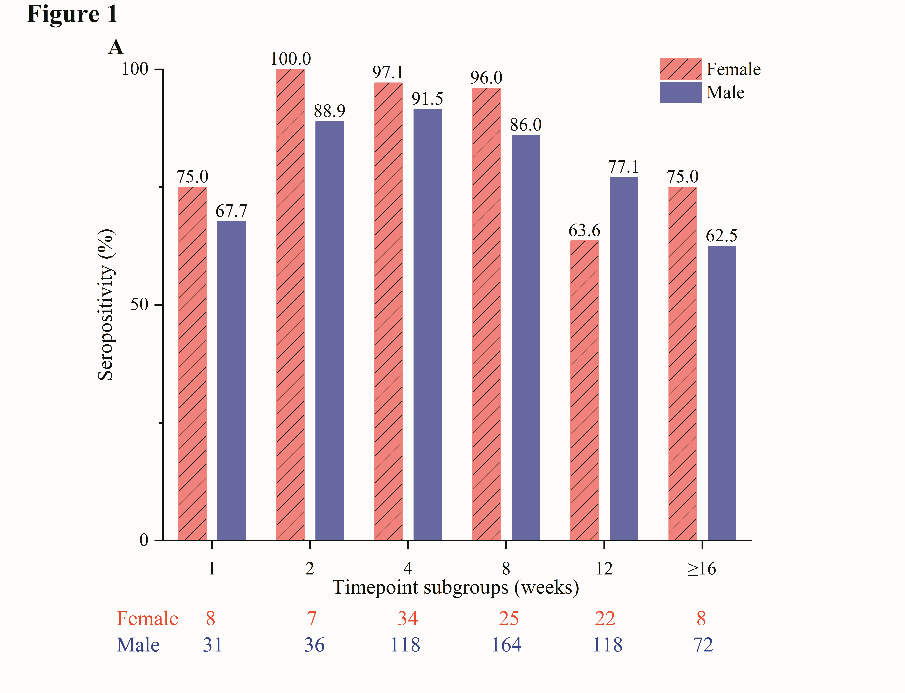 |
| --- |
| 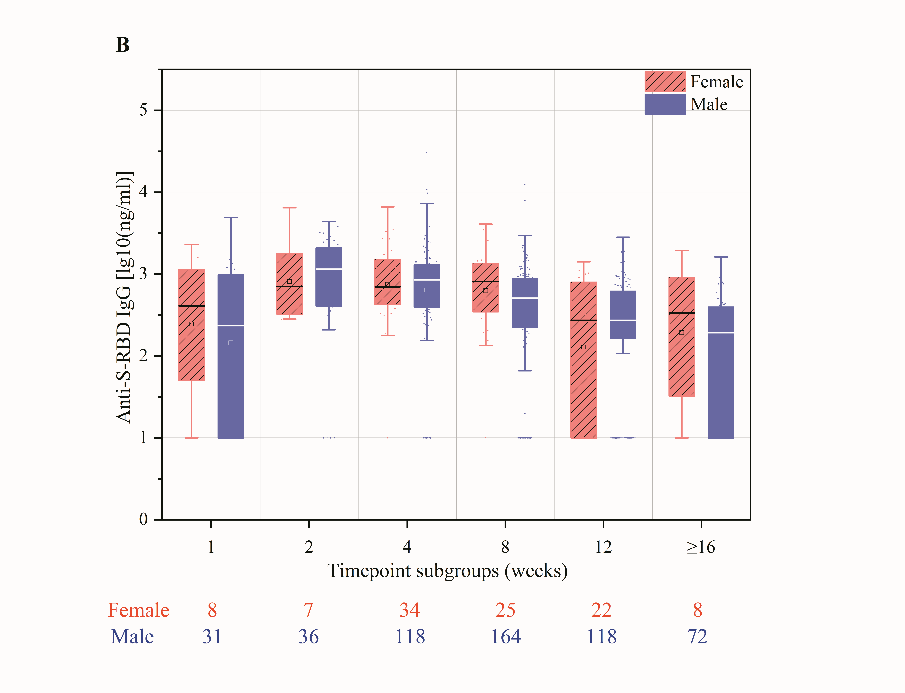 |

**Supplementary Figure 2.** The comparison of antibody response in week 1, 2, 4, 8, 12, ≥16 subgroups according to age. Seropositivity rates (A) and the anti-S-RBD IgG titer (B) at different age subgroups. The week 1, 2, 4, 8, 12, ≥16 subgroups included the samples collected at 7±2 days, 14±2 days, 28±7 days, 56±14 days, 71-98 days and ≥112 days after 2nd vaccination, respectively. *: *p* <0.05.

| 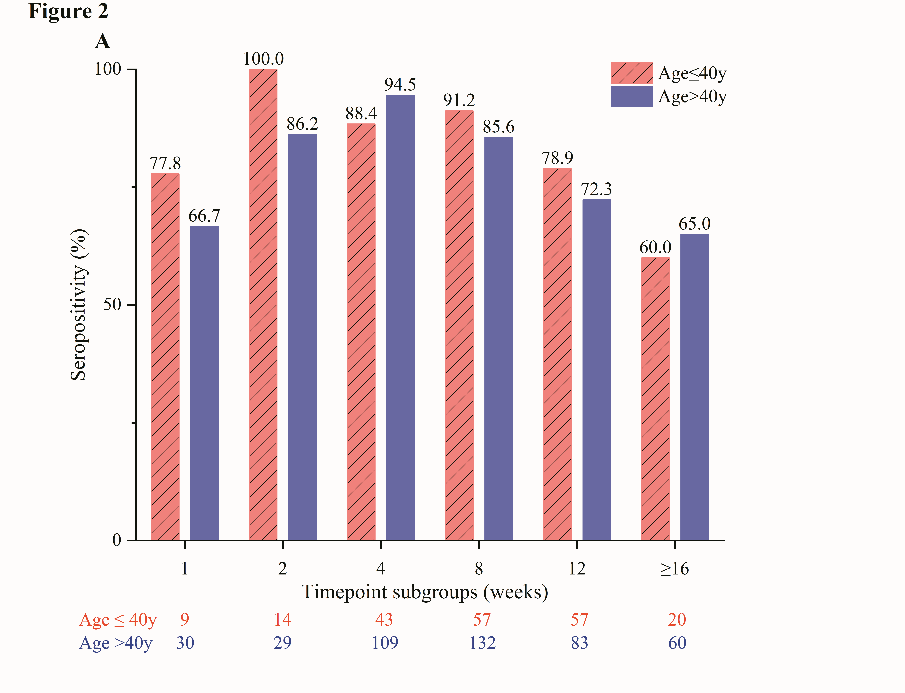 |
| --- |
| 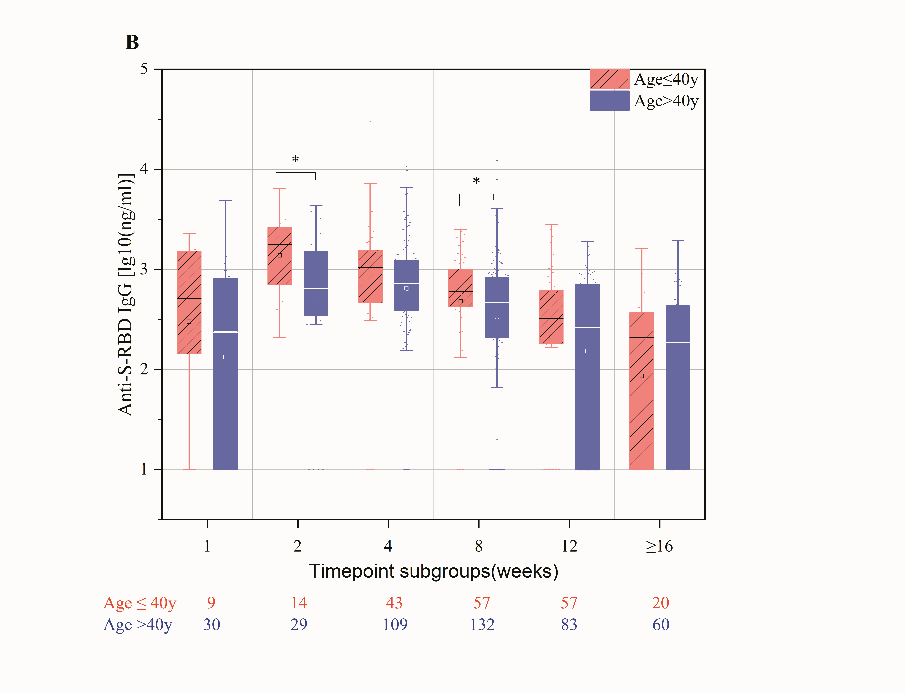 |

**Supplementary Figure 3.** The comparison of antibody response in week 1, 2, 4, 8, 12, ≥16 subgroups according to BMI. Seropositivity rates (A) and the anti-S-RBD IgG titer (B) in normal weight and overweight subgroups (BMI ≥24 kg/m^2^). The week 1, 2, 4, 8, 12, ≥16 subgroups included the samples collected at 7±2 days, 14±2 days, 28±7 days, 56±14 days, 71-98 days and ≥112 days after 2^nd^ vaccination, respectively.

| 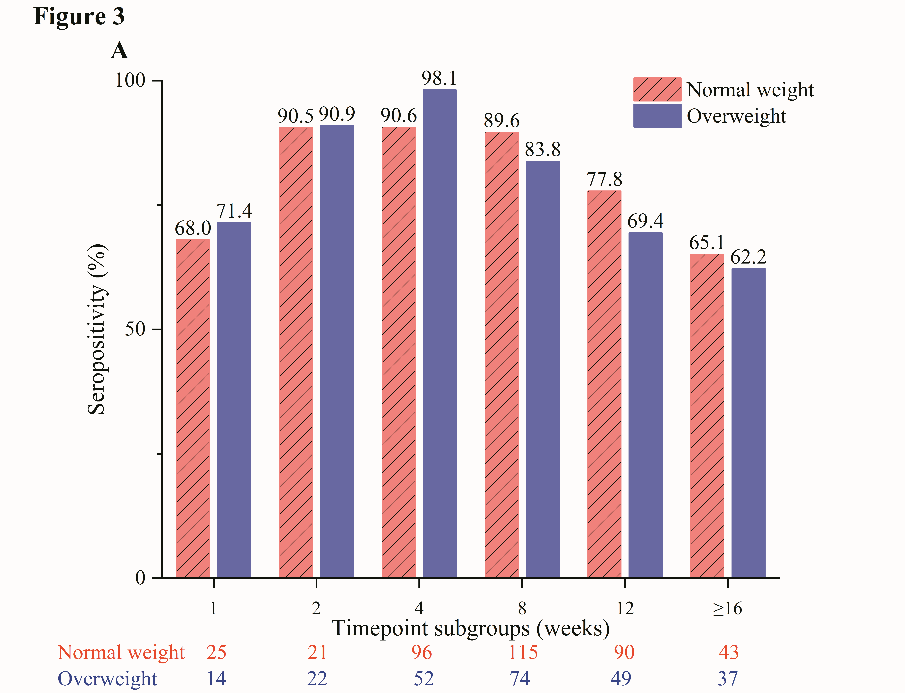  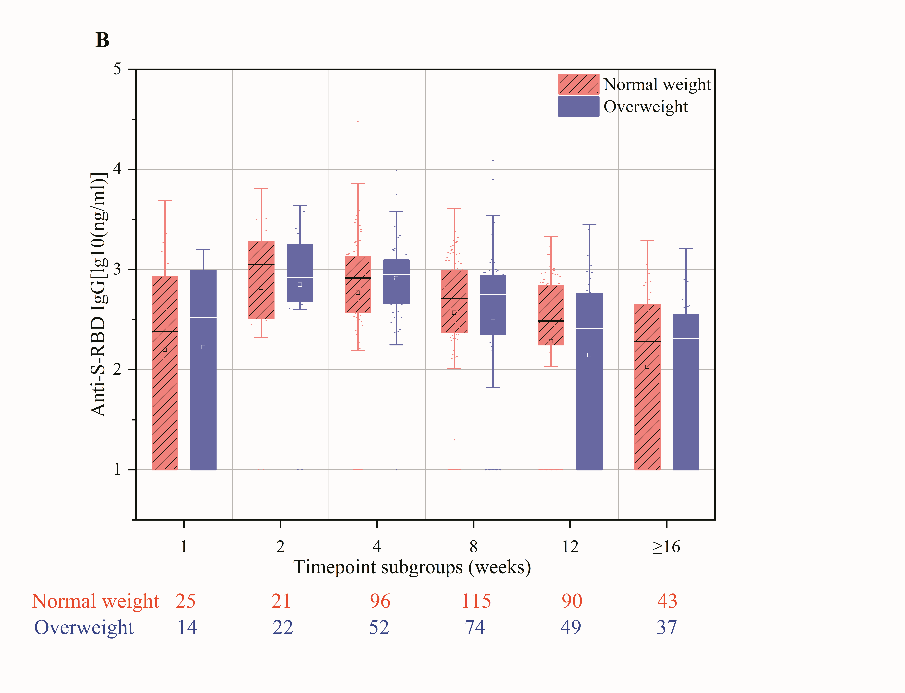 |
| --- |
